# Anteroposterior glide versus rotating platform low contact stress (LCS) knee arthroplasty: a randomised controlled trial

**DOI:** 10.1186/1471-2474-8-87

**Published:** 2007-08-31

**Authors:** Gayle Walley, Sandeep Datir, Murali Sayana, Aziz Rahmatalla, Ian Dos Remedios, Charles Wynn-Jones, Stephen Bridgman, Nicola Maffulli

**Affiliations:** 1 University of Keele, Department of Trauma and Orthopaedics, Keele University Medical School, Thornburrow Drive, Hartshill, Stoke-on-Trent, Staffordshire, ST4 7QB, UK; 2 University Hospital of North Staffordshire NHS Trust, Department of Trauma and Orthopaedics, Newcastle Road, Stoke-on-Trent, ST4 6QG, UK; 3 Newcastle-under-Lyme Primary Care NHS Trust, Department of Public Health, Civic Offices, Merrial Street, Newcastle-under-Lyme, Staffordshire, UK

## Abstract

**Background:**

Fifty thousand knee replacements are performed annually in the UK at an estimated cost of £150 million. Post-operative improvement depends on a number of factors including implant design and patient associated factors. To our knowledge there are no published study's comparing the results of AP glide and rotating platform designs of LCS knee arthroplasty. Therefore we feel that a study is required to investigate and compare the effects of two types of LCS total knee arthroplasty on joint proprioception and range of motion.

**Methods/Design:**

Patients will be randomised to receive either a LCS AP glide or Rotating platform prosthesis. Clinical scores (Oxford knee score, American knee society score, EuroQol), range of motion and proprioception will be assessed prior to and at 3,6, 12 and 24 months after the operation. Proprioception will be assessed in terms of absolute error angle (mean difference between the target angle and the response angle). Knee angles will be measured in degrees using an electromagnetic tracking device, Polhemus 3Space Fastrak that detects positions of sensors placed on the test limb. Student's t-test will be used to compare the mean of two groups.

**Discussion:**

Evidence is lacking concerning the best prosthesis to use for patients undergoing total knee replacement. This pragmatic randomised trial will test the null hypothesis that anteroposterior glide LCS knee arthroplasty does not result in better post operative knee motion and proprioception as compared to rotating platform LCS knee.

**Trial Registration:**

ISRCTN52943804

## Background

Total knee replacement surgery is one of the commoner types of replacement surgeries performed nowadays with about 50,000 knee replacements being performed annually in the UK. Fixed bearing total knee arthroplasty (TKA) has been used as a standard treatment for osteoarthritis of the knee joint. It allows fairly unconstrained motion of the knee joint. Flexion and extension of the knee involve antero-posterior gliding, rotation and femoral rollback, which are not reproduced in fixed bearing TKA.

The LCS (low contact stress) rotating platform TKA system attempts as near-normal reproduction of knee motion as possible, minimising interface stresses by allowing rotation (with this design, the posterior cruciate ligament has to be sacrificed). The AP (antero-posterior) glide LCS type allows both antero-posterior glide and rotation. With this design, the posterior cruciate ligament needs to be intact to allow normal femoral rollback. Rather than imposing a predetermined pattern of motion, the AP glide design permits bearing movement, which corresponds to the requirements of individual patient anatomy. We hypothesise that these features of the AP glide TKA may allow improved joint position sense (proprioception) and better overall functional outcome.

The LCS rotating platform design has good clinical results, but, as results improve in terms of flexion and long term survival, more subtle measures of the return to near normal post-operative function become important. Proprioception is affected by osteoarthritis of the knee joint [1]. As proprioception is one of the protective mechanisms of a joint, one should aim to preserve or enhance it. Proprioceptive function after TKA is debated, with studies reporting conflicting results [2-4]. Post-operative improvement depends on a number of factors, including implant design and patient-associated factors. To our knowledge, there are no published studies comparing the results of AP glide and rotating platform TKA.

## Methods/Design

### Null Hypothesis

Antero-posterior glide LCS knee arthroplasty does not result in better post-operative knee motion and proprioception as compared to rotating platform LCS knee arthroplasty.

### Objectives

The trial proposes to:

1. Study the improvement in knee motion and overall function after AP glide and rotating platform design LCS TKA.

2. Examine the effect of this intervention on proprioception.

### Trial Summary

This study aims to compare the post operative knee motion and proprioception after LCS AP glide and rotating platform knee replacement designs. We propose to recruit a total of 50 patients (25 in each group) in the trial. The patients will be recruited from the pre-operative assessment clinic following their informed consent to take part in the study. Randomisation will take place at the time of surgery. The primary outcome measure will be the knee motion and prorioception. The secondary outcomes will include the Oxford Knee Score [5], American Knee Society Score [6], EuroQol EQ-5D [7], and post-operative complications. All outcomes will be evaluated at 3, 6, 12 and 24 months after the index surgery.

### Study type

A randomised controlled trial comparing the effects of AP glide and rotating platform design LCS TKA.

#### Participants

Participants will be patients undergoing primary bi- (tibio-femoral joint) or tri- (tibio-femoral-patellar joint) compartmental knee arthroplasty. Patients will be recruited from the Orthopaedic Pre-operative clinics held at the Orthopaedic Outpatient Department of the City General Hospital, Stoke on Trent, England.

#### Treatment details

All surgical procedures will be performed in the operating theatres of the City General Hospital, Stoke on Trent, England. In the operating theatre, patients will be randomised to receive one of the two types of prosthesis using a standard surgical technique.

#### Eligibility

Patient will be included if:

1. They require a primary bi- or tri-compartmental knee replacement.

2. They have given their voluntary, written informed consent.

Patients will be excluded if:

1. They are going to have revision knee surgery.

2. They are scheduled to have bilateral knee replacement in one sitting.

3. They suffer from rheumatoid arthritis, diabetes mellitus, posttraumatic arthritis or any form of neurological disorder, which can affect the joint position sense.

4. They have had or will require a major knee arthrotomy on the other same side within 6 months period.

5. They have more than 20° of varus, valgus or flexion deformity

#### Recruitment

Patients will be recruited from the orthopaedic pre-operative assessment clinic. Their medical notes will be screened to assess suitability. At the pre-operative visit, patients will be informed of the nature of the study by their consultant orthopaedic surgeon. Eligible patients will be provided with an information leaflet explaining the nature of the study. They would be invited to ask questions regarding the study. They will then be given the opportunity to think about whether they would like to participate or not. During this period, they will be contacted by the research nurse to discuss their participation and to answer any questions they may have. On the day of the admission, the patients will have a further opportunity to discuss the participation in the trial. Patients willing to take part in this trial will be then invited to sign a consent form by a member of the research team with the approval of the operating consultant. Baseline data will be acquired on admission. Once patients have consented for the study, they will then be randomised in the operating theatre.

#### Randomisation

Patients will be randomised using a minimisation programme called Minim [8]. On arrival in the operating suite, eligibility criteria for each patient will be re-checked. Randomisation will take place once the knee replacement surgery is started and it is decided that either of these two TKA designs could be used. The patient will be allotted a group, i.e. AP glide knee replacement or rotating platform knee replacement. Provision will be made for an easy access to the randomisation service on the operating theatre computer. The patients in both groups will have post-operative rehabilitation regime as prescribed by the operating surgeon. Any complication during or after the operation will be noted. Patients will then be seen at their post operative visit at 3, 6, 12 and 24 months where knee score forms will be completed, proprioception will be evaluated and range of joint movement will be quantified using the FASTRACK system (*Polhemus Fastrack 3Space Manual*, Polhemus Inc, Colchester, VT, 1992).

### Assesment of outcome

All the assessment procedure will be performed in the Bionics Laboratory, City General Hospital, Stoke on Trent, England.

#### Primary outcome

The primary outcome measure for this study will be the improvement in knee motion (as measured by FASTRAK system). This will be done before operation and at 3, 6, 12 and 24 months after the operation. Fastrak has been developed by Polhemus (Colchester, VT, USA) in the early 1990 to monitor aircraft pilots and to be used in the animation industry. FASTRAK is a 3 dimensional tracking device based on emission of a low frequency magnetic field by a transmitter. Within this magnetic field, the position and orientation of up to four sensors and their spatial relationship can be recorded simultaneously. FASTRAK provides dynamic, real-time six degrees of freedom measurement. It computes the position (X, Y, Z Cartesian coordinates) and orientation (azimuth, elevation and roll) of the sensor through space relative to the source transmitter. Each sensor measures data in three different planes: a primary plane of movement and two secondary planes. The recording is performed digitally on a computer. The data can be represented as a real time graph or numerically as range of movement. To perform each Fastrak assessment, double-sided adhesive discs will be used to fix all sensors to the skin. The first sensor will be placed just above the lateral malleolus. The second sensor will be placed just lateral to the the anterior tibial tuberosity of the tibia. The third sensor will be placed over the lateral condyle of the femur. The wires connecting the sensors to the transmitter were secured with Micropore surgical tape (3M Healthcare Ltd. Loughborough, Leicestershire, UK) to avoid pull on the sensors.

Three flexion and extension movements will be performed consecutively without stopping so as to obtain three measurements. The Fastrak will be centred at 0° at full knee extension before movements begin. Each patient will perform a trial run to confirm that the patient satisfactorily understood the instructions. The average range of motion of the three trials will be used for analysis. A preliminary feasibility study showed a variability of 3° ± 1° in the measurements of range of flexion and extension of the knee performed using the Fastrak system in 25 patients on the waiting list for total knee arthroplasty. All measurements will be taken by the same researcher (AR) who is not involved in the clinical management of the patients.

#### Secondary outcomes

1. Proprioception, as measured by absolute error angle, i.e. the difference between actual and perceived angle through which the joint has been moved passively, will be measured using the Fastrak apparatus (Polhemus, Colchester, VT, USA). Briefly, each subject will have their knee screened from view while sitting on an examination couch with the knees hanging from the side. Each knee will be moved passively to one of three predetermined position (full extension, 30° and 60° of flexion), and held in that position for 5 seconds. The limb will then returned to a resting position. The patient is then asked to reproduce the knee position to which it had been moved passively. The test will be repeated three times per each of three positions.

2. The American Knee Society Score [6], Oxford Knee Score [5], and EuroQol EQ-5D [7] will be recorded pre-operatively and at 3,6,12 and 24 months after the operation.

3. Complications.

### Statistical considerations

#### Sample size

The sample size of the trial has been calculated on the basis of a pilot study. Range of motion of the knee joint is the primary outcome in our study. We found that the standard deviation for this parameter in the pilot study was 20°. The anticipated difference between the mean (which we think would be of clinical significance) was considered as 20°. The sample size required for an alpha of 0.05 and a power of 90% was found to be 22 cases per group. To allow for dropouts, recruitment a total of 25 patients in each group of the study would be a reasonable option.

#### Analysis

*t *test will be used to compare variables between the groups where the data are approximately normally distributed, possibly after transformation. Non-normal data will be assessed using the Mann-Whitney test. Any imbalances between the groups at baseline will be adjusted for by using ANCOVA. Analysis will be performed using the intention to treat criterion.

#### Blinding

The laboratory personnel will be blinded to the treatment allocation, and will perform knee motion and proprioceptive assessments. The research nurse, who will be blinded to the treatment allocation, will perform the knee scoring of both groups.

#### Data collection

The data will be collected from the medical records during the in hospital stay of the patients. Final data collection will take place at the post-operative visit at 24 months. The last observation will be carried forward for data analysis.

#### Protocol deviations

Any changes to the protocol will be recorded. Any amendments that may be required once the trial has started will be submitted for approval by the ethics committee.

#### Informed consent

All eligible patients who agree to take part will sign an informed consent form. They will confirm that they have been given the information that they require, and that the study has been explained to them.

#### Confidentiality

Only the trial organisers/administrators will have access to patients' notes and questionnaires. All recorded information will be stored separately from patients' names and addresses.

#### Ethics

The trial has been given ethics permission by our Local Ethics Committee, and is registered as ISRCTN 52943804.

## Discussion

Evidence is lacking concerning the best prosthesis to use for patients undergoing total knee replacement. This pragmatic randomised trial will test the null hypothesis that anteroposterior glide LCS knee arthroplasty does not result in better post-operative knee motion and proprioception as compared to rotating platform LCS knee.

## Competing interests

The author(s) declare that they have no competing interests.

## Authors' contributions

GW, SD, MS, CWJ, SB and NM conceived the study. GW, SD and MS performed the review of the literature and wrote the initial protocol. They also filled in the Research and Development forms and the Ethics Committee Application forms. IDR and CWJ advised on the practicalities of the surgery. AR advised on the proprioception and range of motion measurements. SB advised on the design of the study and the statistical analysis. All authors read and approved the final manuscript.

## Pre-publication history

The pre-publication history for this paper can be accessed here:


